# Differential Effects of IL-12 on Tregs and Non-Treg T Cells: Roles of IFN-γ, IL-2 and IL-2R

**DOI:** 10.1371/journal.pone.0046241

**Published:** 2012-09-27

**Authors:** Jingxian Zhao, Jincun Zhao, Stanley Perlman

**Affiliations:** 1 Department of Microbiology, University of Iowa, Iowa City, Iowa, United States of America; 2 Institute for Tissue Transplantation and Immunology, Jinan University, Guangzhou, Guangdong, China; New York University, United States of America

## Abstract

Complex interactions between effector T cells and Foxp3^+^ regulatory T cells (Treg) contribute to clinical outcomes in cancer, and autoimmune and infectious diseases. Previous work showed that IL-12 reversed Treg-mediated suppression of CD4^+^Foxp3^−^ T cell (Tconv) proliferation. We and others have also shown that Tregs express T-bet and IFN-γ at sites of Th1 inflammation and that IL-12 induces IFN-γ production by Tregs *in vitro*. To investigate whether loss of immunosuppression occurs when IFN-γ is expressed by Tregs we treated mouse lymphocyte cultures with IL-12. IFN-γ expression did not decrease the ability of Tregs to suppress Tconv proliferation. Rather, IL-12 treatment decreased Treg frequency and Foxp3 levels in Tregs. We further showed that IL-12 increased IL-2R expression on Tconv and CD8 T cells, diminished its expression on Tregs and decreased IL-2 production by Tconv and CD8 T cells. Together, these IL-12 mediated changes favored the outgrowth of non-Tregs. Additionally, we showed that treatment with a second cytokine, IL-27, decreased IL-2 expression without augmenting Tconv and CD8 T cell proliferation. Notably, IL-27 only slightly modified levels of IL-2R on non-Treg T cells. Together, these results show that IL-12 has multiple effects that modify the balance between Tregs and non-Tregs and support an important role for relative levels of IL-2R but not for IFN-γ expression in IL-12-mediated reversal of Treg immunosuppression.

## Introduction

Natural regulatory T cells (Tregs), identified by the expression of Foxp3, play an important role in down-regulating immune responses and an imbalance between numbers of Tregs and conventional CD4 (Tconv) and CD8 T cells contributes to outcomes in cancer and autoimmune and infectious diseases [1]–[4]. Interactions between Tregs and non-Tregs result in modifications of the function of both cell types, with the goal of minimizing detrimental side effects of pro-inflammatory immune responses while maximizing the efficacy of processes such as pathogen clearance. Further, the function of both cell types and the balance between the pro- and anti-inflammatory immune responses may be affected by cytokines and other molecules produced by cells such as dendritic cells, in the inflammatory milieu.

While Tregs are immunosuppressive at sites of inflammation, Tregs may express transcription factors and cytokines that parallel those of Tconv cells. Thus, Tregs express T-bet and IRF4 at sites of Th1 and Th2 inflammation, respectively [5]–[7]. Additionally, we and others have shown that Foxp3^+^ Tregs express IFN-γ during infections [5], [8]. Unfractionated populations of Tregs harvested from inflammatory sites, which include cells expressing IFN-γ, remain immunosuppressive. Suppression can be demonstrated in T cell proliferation assays after stimulation with either anti-CD3 mAb or virus-specific peptides [8]. In contrast, other studies suggest that a pro-inflammatory milieu may result in diminished Treg suppressive function [9]–[11]. In a model of experimental autoimmune encephalomyelitis (EAE), CNS-derived epitope MOG_35–55_-specific Tregs showed reduced ability to inhibit proliferation of Tconv of the same specificity isolated from the inflamed CNS [9]. This reduction in inhibitory function was IL-6 and TNF-dependent.

IL-12 has also been shown to enhance activation and proliferation of Tconvs even if Tregs are present, possibly reflecting an IL-12-mediated reduction in Treg suppressive function [12]. In other studies, IL-12 was shown to induce IFN-γ production by Tregs *in vitro* and *in vivo* [5], [13]; IFN-γ expression by Tregs may indicate that these cells are transiting to Th1 effector cells [13]. Thus, in apparent conflict with the results using suppression assays, these data suggest that IFN-γ expression is a marker for reduced immunosuppressive ability. However whether IFN-γ expression actually affects Treg suppressive function has not been assessed experimentally. Collectively these results suggest that IL-12 has independent effects on Tconv and CD8 T cells as opposed to Tregs. Consequently, the effect of IL-12 (or other cytokines) on the T cell response in a total lymphocyte culture will reflect the summation of effects on the different cell types.

In order to delineate the roles of these cytokines, particularly IL-12, on Treg function, we treated lymphocytes harvested from spleens and lymph nodes of naïve mice with a panel of cytokines. Treatment with IL-12 induced IFN-γ expression by both Tconv and Tregs and reduced Treg frequency and Foxp3 expression. We also demonstrated that IFN-γ^+^ Tregs are as immunosuppressive as IFN-γ^-^ Tregs. Our results showed that IL-12 functioned, in part, by diminishing IL-2 production by Tconv and CD8 T cells in the mixed cultures and by down-regulating IL-2 receptor expression on Tregs but up-regulating it on non-Treg T cells.

## Materials and Methods

### Ethics Statement

This study was carried out in strict accordance with the recommendations in the Guide for the Care and Use of Laboratory Animals of the National Institutes of Health. Mice were housed in the Animal Care Facility at the University of Iowa. The protocol was approved by the University of Iowa Animal Care and Use Committee (Protocol Number: 1007161). All efforts were made to minimize animal suffering.

### Mice

Specific pathogen-free C57BL/6 (B6) and B6/Thy1.1 mice were purchased from the National Cancer Institute, Bethesda, MD. *Foxp3^gfp^* mice on a B6 background were kindly provided by Dr. A. Rudensky (Sloan-Kettering Institute) and were bred to a Thy1.1 background. IL-12Rβ2^−/−^ mice on a B6 background were obtained from Dr. J. Harty (University of Iowa).

### Cytokines, Chemicals, Antibodies and other Reagents

Recombinant mouse IL-12 and IFN-γ (R&D Systems, Minneapolis, MN), mouse IL-27 (Biolegend, San Diego, CA), human IL-6 (GIBCO, Invitrogen, Grand Island, NY) and human IL-2 (Biological Resources Branch, NCI-Frederick, NIH) as well as CpG (ODN1668, Sigma, St. Louis, MO) and LPS (R-form, ALEXIS Biochemicals, Farmingdale, NY) were obtained from the indicated vendors. The following antibodies and reagents were used: CD4-FITC, -PE or -PerCP-Cy5.5 (RM 4–5), IL-2-PE (JES6-5H4), CD25-PE or -APC (PC-61),CD122-PE(5H4), PE Annexin V Apotosis Detection Kit all from BD Biosciences (San Diego, CA); purified functional anti-CD3 (145-2C11), CD4-PE-Cy (RM 4–5), CD8-APC or -PE-Cy-7 (53–6.7), Foxp3-FITC, -PE or -Alexa Fluor 647 (FJK-16s), T-bet-PE (eBio4B10), IFN-γ-PE or -APC (XMG1.2), all from eBioscience (San Diego, CA).

### Lymphocyte Cultures

Lymphocytes prepared from lymph nodes and/or spleens of mice were stimulated with soluble anti-CD3 mAb (0.5 µg/ml) in the presence of various cytokines. Unless indicated, cells were cultured at 1×10^6^ cells/ml in 24-well tissue culture plates (2 ml/well) and IL-12 was used at 1 ng/ml. To analyze cell proliferation, cells were labeled with CFSE (2 µM, Invitrogen) before culture. To detect intracellular IFN-γ or IL-2 production by T cell subpopulations, PMA (50 ng/ml, Sigma), ionomycin (1 µg/ml, Sigma) and Golgi^plug^ (1 µl/ml, BD Biosciences) were added for the last 4 hr of culture.

### Treatment with TLR Agonists

5x10^6^ B16-Flt3L cells [14] (obtained from Dr. J. Harty) were inoculated subcutaneously into 12-wk old B6 mice. Twelve to fourteen days post inoculation, spleens were harvested, digested with collagenase and DCs were isolated using anti-CD11c microbeads (Miltenyi Biotec, Auburn, CA). T cells were enriched from spleens of B6 or IL-12Rβ2^−/−^ mice using a Pan T Cell Isolation Kit II (Miltenyi Biotec). 1×10^5^ T cells were cultured with 1×10^4^ DCs in the presence of anti-CD3 mAb and medium or IL-12 or LPS (1 µg/ml) or CpG (1 µM) for 72 hr in a 96-well round-bottom plate. In parallel wells, 2×10^5^ unfractionated lymphocytes were treated under the same conditions. Samples were analyzed in triplicate.

### In vitro Suppression Assays

To evaluate the function of IFN-γ producing Tregs, lymphocytes were prepared from *Foxp3^gfp^*/Thy1.1 mice and stimulated with anti-CD3 mAb in the presence of IL-12 or medium as control. After 66 hr, cells were harvested, washed and enriched for CD4^+^ T cells using a CD4 T Cell Isolation Kit II (Miltenyi Biotech). GFP^+^ Tregs were then sorted. Approximately >90% and <3% of Tregs expressed IFN-γ in the IL-12 and medium groups, respectively. Responder Tconvs were isolated from naïve *Foxp3^gfp^* (Thy1.2) mice. For suppression assays, Tregs were co-cultured with CFSE-labeled (2.5 µM) responder Tconvs at the indicated ratios (Tregs plus responders  = 5×10^4^ cells/well) in 96-well round bottom plates in the absence of IL-12. Wells also contained 2×10^5^ T-cell depleted splenocytes (irradiated at 2500 rad) and anti-CD3 mAb. After 66 hr, Thy1.1^−^ Thy1.2^+^ Tconv cells were analyzed for CFSE dilution by flow cytometry.

To evaluate the function of Tregs in the presence of IL-12, responder T cells were enriched from naïve B6/Thy1.1 mice using a Pan T cell Isolation Kit II, and Tregs were isolated from B6 mice using a CD4^+^CD25^+^ Regulatory T cell Isolation Kit (Miltenyi Biotec). For suppression assays, Tregs were mixed with CFSE-labeled (2.5 µM) responder T cells at the indicated ratios (Tregs plus responders = 5×10^4^ cells/well). Cells were cultured in the presence of 2×10^5^ irradiated splenocytes and anti-CD3 mAb with or without IL-12 in 96-well round bottom plates. After 66 hr, Thy1.1^+^ Tconvs and Thy1.1^+^ CD8 T cells were analyzed for CFSE dilution by flow cytometry. The Division Index (DI) was obtained using FlowJo software (Tree Star, Inc., Ashland, OR). A normalized DI was calculated as follows: % normalized DI = 100% × (DI of responders plus Tregs/DI of responders only).

### Flow Cytometry

A Foxp3 Staining Buffer Set (eBioscience) was used for Foxp3 or T-bet staining or when cells were analyzed for Foxp3 and cytokine expression simultaneously; otherwise, BD Cytofix/Cytoperm and Perm/Wash buffers (BD Biosciences) were used in intracellular cytokine staining assays. Cell sorting was performed with a FACSDiva or FACSAria and cell analysis with a FACSCalibur or LSRII (BD Biosciences).

### ELISA

Lymphocytes from lymph nodes of B6 mice were stimulated with anti-CD3 mAb in the presence of IL-12 (1 ng/ml) or IFN-γ (100 ng/ml) in 24-well plates as described above. Supernatants were collected at 48 hr after culture. IL-2 ELISAs were performed using reagents and protocols provided by the manufacturer (eBioscience, Mouse IL-2 ELISA Ready-SET-Go kit). Samples were plated in duplicate.

### Statistics

Data are presented as means ± standard errors of the means (SEM). Differences between two groups were determined by Student’s two-tailed unpaired *t* tests or paired *t* tests (when normalized DI were compared), using GraphPad Prism 5.03 software. Differences with values of *P*<0.05 were considered significant. *, *P*<0.05; **, *P*<0.01; ***, *P*<0.001.

## Results

### IL-12 Induces IFN-γ Expression by Tregs and Inhibits Treg Proliferation and Foxp3 Expression

We and others showed that Tregs express IFN-γ at sites of inflammation and can be induced to express IFN-γ *in vitro* [5], [8], [13]. In specific, IFN-γ is expressed by virus-specific Tregs in the CNS of mice infected with the rJ2.2 strain of mouse hepatitis virus (MHV) [8]; we used information obtained from this experimental system as the basis for the approach described here. To determine whether cytokines had a role in IFN-γ expression by Tregs, we initially focused on cytokines that are up-regulated in the CNS of MHV-infected mice and remain elevated as the infection resolves [15]. Among these cytokines, IL-12, IL-6 and IFN-γ have roles in T cell polarization and Treg development [16]–[20], so we first evaluated their participation in Treg-mediated IFN-γ expression. Lymphocytes were prepared from the lymph nodes of naïve B6 mice, labeled with CFSE and treated with each cytokine and anti-CD3 mAb for 66 hours. Cells were then stimulated for 4 hours with PMA and ionomycin and evaluated for T cell proliferation and IFN-γ expression.

**Figure 1:**
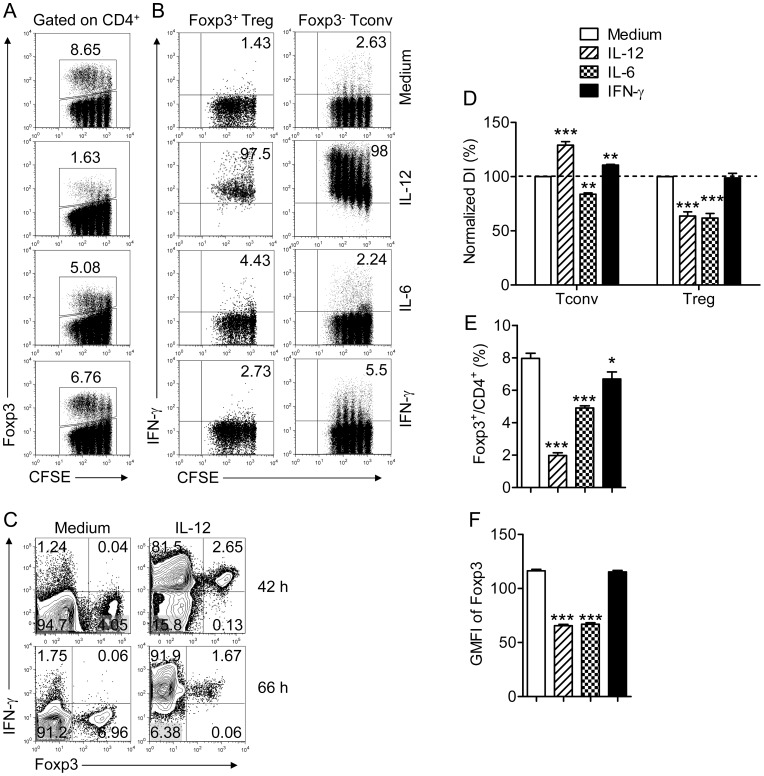
IL-12, but not IL-6 or IFN-γ induces IFN-γ expression by Foxp3^+^ Tregs. Lymphocytes were prepared from lymph nodes of naïve B6 mice, labeled with CFSE and stimulated with anti-CD3 mAb and the indicated cytokine for 66 hr. Cells were then stimulated with PMA and ionomycin for 4 hr. (A) Percentage and CFSE dilution of Foxp3^+^ Tregs in the CD4 T cell population. (B) IFN-γ expression by Tconvs (Foxp3^−^) or Tregs (Foxp^+^) after exposure to IL-12 (1 ng/ml), IL-6 (25 ng/ml) or IFN-γ (100 ng/ml). (C) IFN-γ expression by Tconvs (Foxp3^−^) and Tregs (Foxp^+^) after exposure to IL-12 for 42 or 66 hr. (D) Division indices (DI) were calculated for Tconvs and Tregs and normalized to the groups treated with medium. Data are from three independent experiments. (E) Percentage of CD4 T cells expressing Foxp3 after treatment. Data were acquired from 4–9 independent experiments. (F) Geometric mean fluorescent intensity (GMFI) of Foxp3 in Tregs after indicated treatment, measured in triplicate. Data are from one experiment representative of 3 independent experiments. *P<0.05, **P<0.01, ***P<0.001 compared to cells exposed to medium, Student’s two-tailed paired *t* tests (D) or un-paired *t* tests (E, F).

As shown in Fig. 1B, IL-12 but not IL-6 or IFN-γ induced IFN-γ production by Tregs and Tconvs although IL-12 induced lower levels of IFN-γ in Tregs than in Tconvs. After 42 hours, the majority of Tconv and Tregs expressed IFN-γ; by 66 hrs nearly all of the cells were IFN-γ^+^ (Fig. 1C). Further, IL-12 treatment increased proliferation of Tconvs (Fig. 1B, D) and CD8 T cells (data not shown), but, in contrast, diminished frequency and proliferation of Tregs (Fig. 1A, D, E). IL-12 also decreased levels of Foxp3 in Tregs (Fig. 1F). The net result was a decreased proportion of Tregs expressing reduced levels of Foxp3 in IL-12-treated cell cultures (Fig. 1A, E). IL-6 reduced both Tconv and Treg proliferation although the effects on Treg proliferation were greater (Fig. 1B, D).

**Figure 2:**
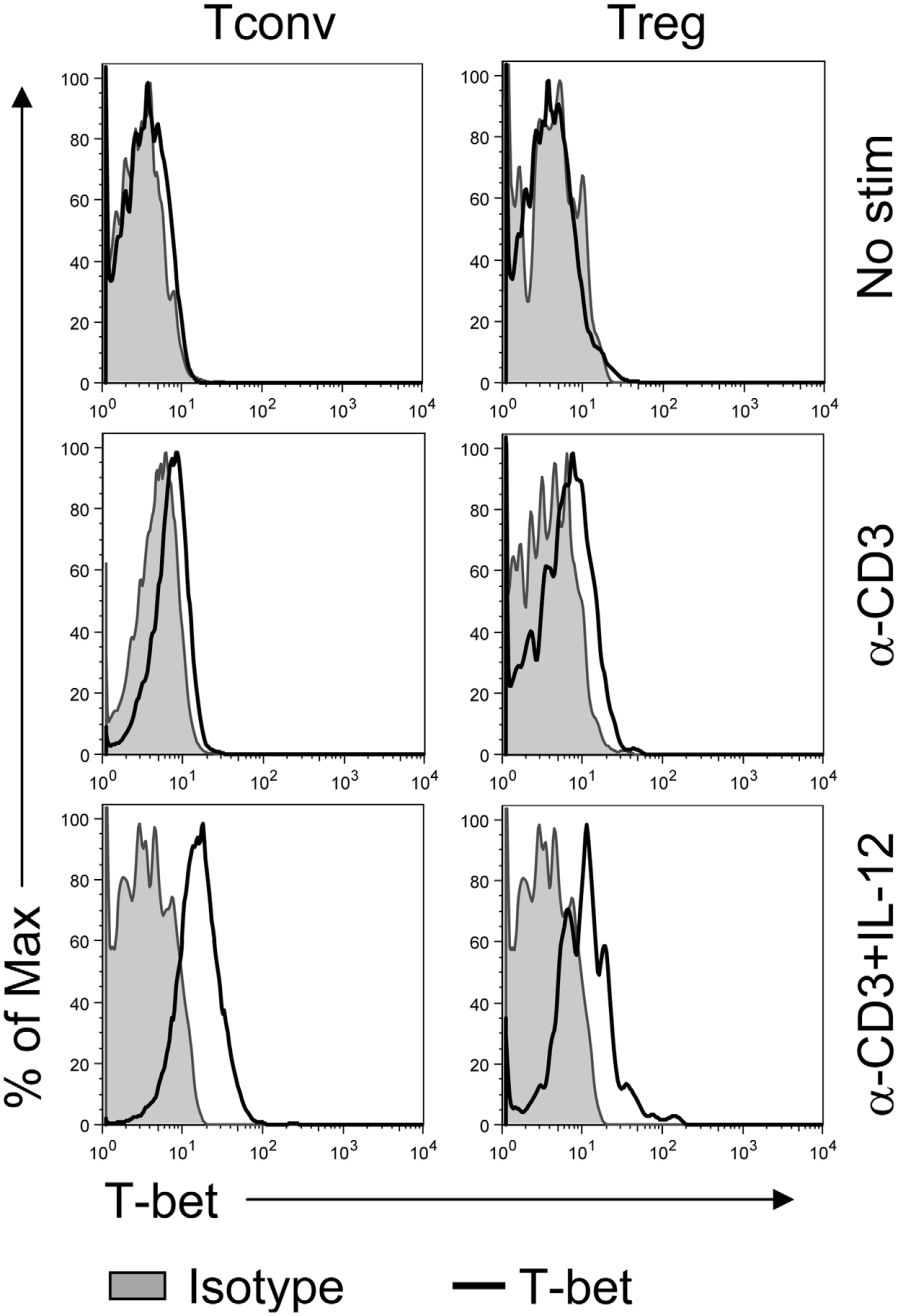
IL-12 induces T-bet expression by Tconvs and Tregs. Lymphocytes were prepared as in Figure 1 and stimulated with anti-CD3 mAb for 66 hr in the presence or absence of IL-12 or not stimulated. T-bet expression in Tconvs and Tregs was measured by flow cytometry. Data are from one experiment representative of 3 independent experiments.

IL-12 treatment increases expression of T-bet, a transcription factor associated with Th1 development [19], [20]; we and others previously showed that T-bet was up-regulated in Tregs localized to sites of Th1-type inflammation [5], [7], [8]. Consistent with these results, T-bet was also increased in Foxp3^+^ Tregs as well as in Tconvs after IL-12 treatment (Fig. 2).

### Treatment with TLR Agonists LPS and CpG Augments IFN-γ Expression by Tregs in an IL-12 Dependent Manner

**Figure 3:**
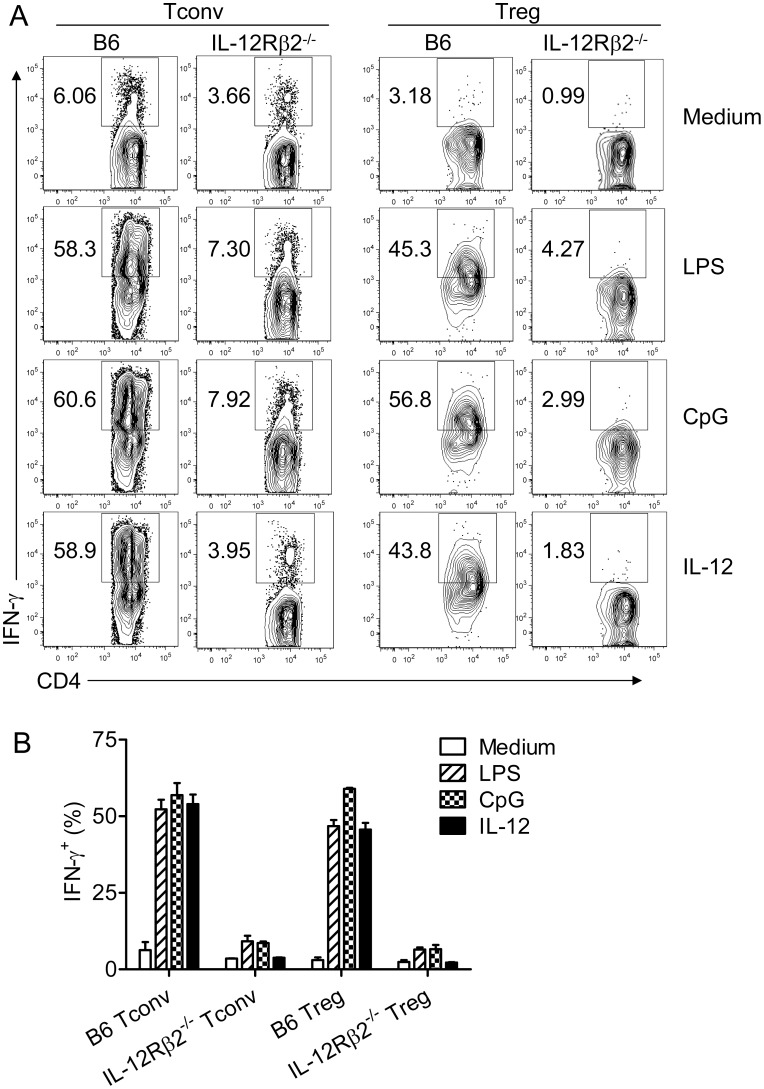
TLR agonists induction of IFN-γ expression by Tconvs and Tregs is IL-12-dependent. Purified T cells were prepared from naïve B6 and IL-12Rβ2^−/−^ mice and mixed with splenic DCs as described in Materials and Methods. Cells were stimulated for 72 hr with anti-CD3 mAb and LPS (1 µg/ml), CpG (1 µM), IL-12 or medium. LPS and CpG induced IFN-γ expression by B6 but not IL-12Rβ2^−/−^ Tconvs and Tregs. (A) Representative FACS plots are shown. (B) Summary data combined from three independent experiments are shown.

To examine the role of IL-12 in IFN-γ induction in Tregs when inflammation is induced by a more physiological mechanism, we treated samples with TLR agonists, LPS or CpG. T cells (B6 or IL-12Rβ2^−/−^) and splenic DCs (B6) were isolated and incubated together in the presence of anti-CD3 mAb and LPS or CpG. The fraction of Tregs expressing IFN-γ increased after LPS or CpG exposure and was similar to levels obtained in cultures treated with IL-12 (Fig. 3). LPS and CpG likely functioned via effects on DCs because no IFN-γ was induced when unfractionated lymph node-derived lymphocytes were treated with LPS or CpG in the absence of added DCs (data not shown). CpG or LPS-mediated augmentation in the proportion of cells expressing IFN-γ was largely dependent on IL-12, since the fraction of IFN-γ^+^ Tregs increased only slightly in cultures containing IL-12Rβ2^−/−^ T cells (Fig. 3). Of note, IL-12Rβ2^−/−^ mice are deficient in IL-12 but not IL-23 signaling [21].

### Treg-mediated Suppression is Reduced by IL-12 but not as a Consequence of IFN-γ Expression

**Figure 4:**
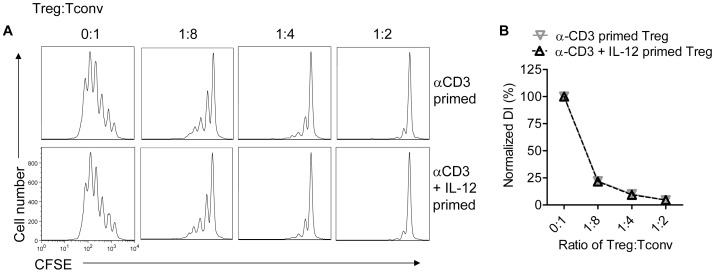
IFN-γ^-^ and IFN-γ^+^ Tregs are equivalently suppressive. Lymphocytes from naïve *Foxp3^gfp^* mice were treated with anti-CD3 with or without IL-12 for 66 hr. Tregs were sorted and co-cultured with CFSE-labeled Tconvs at the indicated ratios for 66 hr in the presence of irradiated splenocytes and anti-CD3 mAb. Tconvs were then analyzed for CFSE dilution. Samples were analyzed in triplicate. (A) Representative histograms are shown. (B) Normalized DI were calculated for each group. Data are from one experiment representative of 3 independent experiments.

Mixed populations of IFN-γ^+^ and IFN-γ^-^ Tregs are able to suppress proliferation of both anti-CD3 mAb and MHV peptide-stimulated T cells [8]. However, it was not possible from these experiments to determine whether IFN-γ expression attenuated suppressive function. To address this question, we treated spleen and lymph node-derived lymphocytes isolated from *Foxp3^gfp^* mice with anti-CD3 mAb in the presence or absence of IL-12. GFP^+^ Tregs were then sorted from the mixed cultures. In the presence of IL-12, >90% of all GFP^+^ cells expressed IFN-γ while 90–98% IFN-γ^-^ Tregs were detected in cultures treated with anti-CD3 mAb alone. Both sets of Tregs (IFN-γ^+^ or IFN-γ^-^) were then mixed with CFSE-labeled Tconvs. Cells were then stimulated with anti-CD3 antibody in the absence of IL-12. As shown in Fig. 4, IFN-γ^+^ and IFN-γ^-^ Tregs inhibited the proliferation of CFSE-labeled Tconv to the same extent.

**Figure 5:**
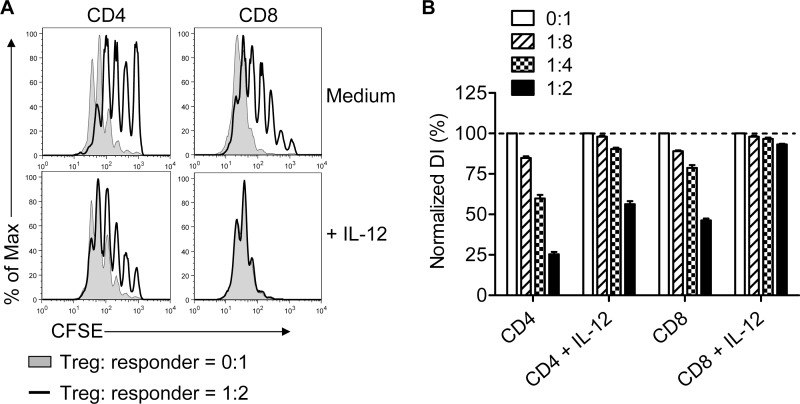
Treg-mediated suppression is reduced in the presence of IL-12. Tregs were isolated from naïve B6 mice and mixed with CFSE-labeled T cells at the indicated ratios. Cells were cultured for 66 hr in the presence of irradiated splenocytes and anti-CD3 mAb with or without IL-12. Samples were analyzed in triplicate. (A) Representative histograms are shown after gating on CD4 or CD8 T cells. (B) DI were calculated and normalized to samples incubated in the absence of Tregs in each group. Data are from one experiment representative of 3 independent experiments.

In these assays, we compared equal numbers of IFN-γ^+^ and IFN-γ^-^ Tregs for suppressive function. However, results in Fig. 1 showed that the numbers of Tregs and their relative expression of Foxp3 were diminished in IL-12-treated cultures. Further, a previous report showed that Treg suppressive function was alleviated by IL-12 [12]. To reconcile these disparate results, we assessed Treg suppression in the presence of IL-12 using Tregs and responder T cells enriched from lymph nodes and spleens. Responder T cells were labeled with CFSE and co-cultured with unlabeled Tregs at several cell ratios. We observed diminished suppression of CFSE-labeled CD4 T cells in the presence of IL-12, in agreement with published results (Fig. 5). Suppression of CD8 T cell proliferation was almost completely abrogated if IL-12 was present in the culture.

### IL-12 does not Induce Apoptosis of Tregs or Treg Conversion

**Figure 6:**
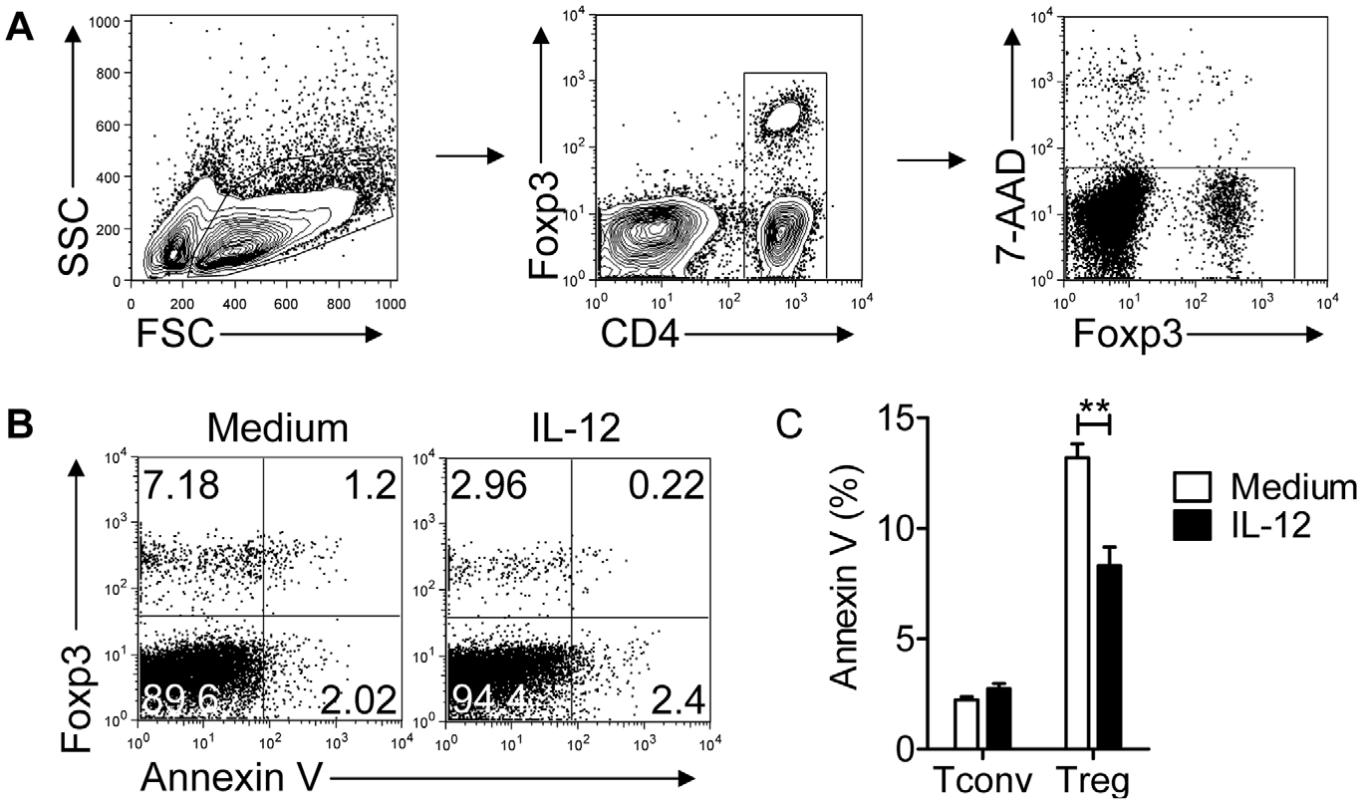
IL-12 does not induce apoptosis in Tconvs or Tregs. Lymphocytes were prepared from *Foxp3^gfp^* mice and cultured in the presence of anti-CD3 mAb with or without IL-12 for 72 hr. (A) Gating strategy to identify live CD4 T cells is shown. (B) Representative FACS plots are shown. (C) Summary of data from 3 independent experiments, each performed in triplicate, is shown. ***P<0.01, Student’s two-tailed un-paired *t* tests.

Treg proliferation was diminished in the presence of IL-12, resulting in an effective change in ratio of the two cell types within a single culture (Fig. 1C, D). However, it is also possible that IL-12 augmented Treg cell death or induced conversion of Tregs to Tconvs, which would have the same effect. To address the role of apoptosis, we measured AnnexinV expression on Tconvs and Tregs in lymph nodes cultures. IL-12 treatment did not increase the percentage of either AnnexinV^+^ Tregs or Tconvs in the cultures, demonstrating that there was no preferential Treg death (Fig. 6).

**Figure 7:**
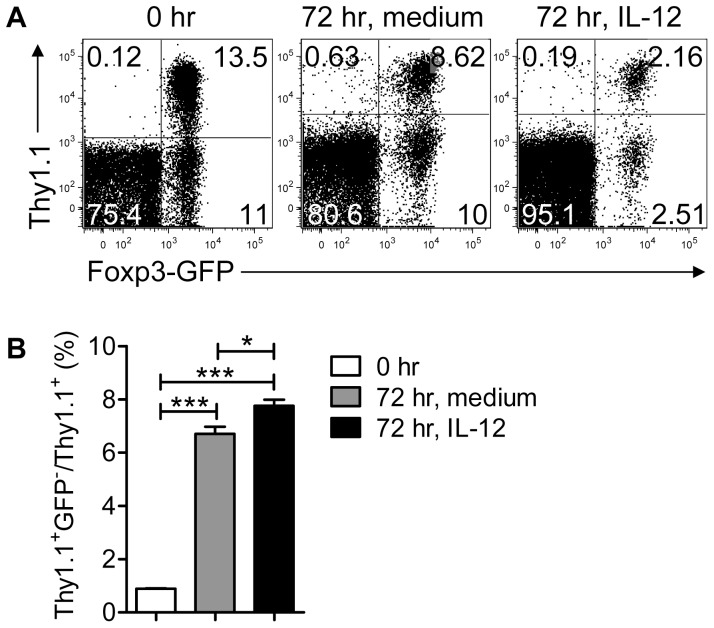
IL-12 does not induce substantial Treg conversion. Tregs were sorted from *Foxp3^gfp^*/Thy1.1 mice and mixed with lymphocytes harvested from naïve *Foxp3^gfp^* mice (Thy1.2). Samples were incubated for 72 hr with anti-CD3 mAb in the presence or absence of IL-12. (A) Representative FACS plots are shown after gating on CD4 T cells. (B) Summary of data showing GFP^-^ fraction of Thy1.1^+^ cells. Data are from 3 independent experiments, each performed in triplicate. *P<0.05, ***P<0.001, Student’s two-tailed un-paired *t* tests.

Loss of Foxp3 expression by Tregs has been demonstrated *in vivo*, although whether this represents Treg instability is controversial [22]–[24]. IL-12 induces IFN-γ expression by Tregs (Fig. 1B); if IL-12 also mediated Treg conversion, this would contribute to a decrease in Treg, and an increase in Tconv numbers. To examine this possibility, *Foxp3^gfp^*/Thy1.1 Tregs were purified from naïve spleens and incubated with unfractionated lymphocytes from *Foxp3^gfp^*/Thy1.2 mice in the presence of anti-CD3 mAb and IL-12 or media. As shown in Fig. 7, IL-12 did not induce an appreciable level of Treg (Foxp3^+^) to Tconv (Foxp3^−^) conversion when compared to samples exposed to medium alone.

### IL-12 Inhibits IL-2 Expression by Tconv and CD8 T Cells and Differentially Affects IL-2R Expression by Tregs and Non-Treg T Cells

**Figure 8:**
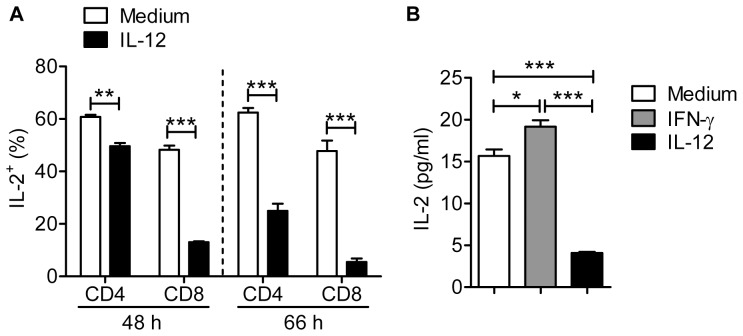
IL-12 inhibits IL-2 expression by T cells. Lymphocytes were prepared from naïve *Foxp3^gfp^* mice and treated with anti-CD3 mAb and IL-12 or IFN-γ. (A). The fraction of CD4 and CD8 T cells expressing IL-2 was diminished after 48 and 66 hr. Data are from 3–5 independent experiments. (B) Levels of IL-2, measured by ELISA, were reduced in IL-12 but not IFN-γ-treated cultures at 48 hr. Data are representative of 2 independent experiments performed in triplicate for stimulation, and each sample plated in duplicate for ELISA. *P<0.05, ***P<0.001, Student’s two-tailed un-paired *t* tests.

**Figure 9:**
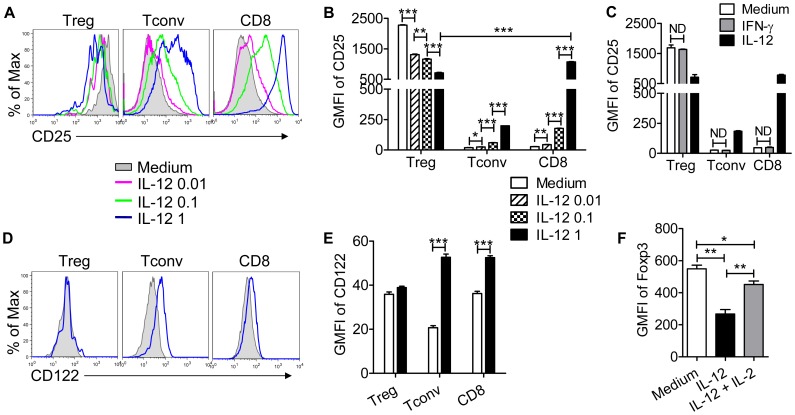
IL-12 has opposite effects on IL-2R expression by Tregs vs non-Treg T cells. Lymphocytes were prepared from naïve *Foxp3^gfp^* mice and treated with anti-CD3 mAb and IL-12 or IFN-γ for 66 hr. (A, B) CD25 expression was decreased on Tregs and increased on Tconvs and CD8 T cells by IL-12 in a dose-dependent fashion. Representative histograms are shown in (A) and summary data are shown in (B). (C) IFN-γ did not affect CD25 expression by Tregs and non-Treg T cells (ND-no difference). (D, E) IL-12 increased CD122 expression on Tconvs and CD8 T cells but not on Tregs. Representative histograms are shown in (D) and data are summarized in (E). (F) IL-12 diminished Foxp3 GMFI in Tregs and this was largely reversed by IL-2 (100 U/ml) treatment. Data are representative of three independent experiments performed in triplicate. *P<0.05, **P<0.01, ***P<0.001, Student’s two-tailed un-paired *t* tests.

Treg development, homeostasis and suppressive function are dependent upon IL-2 [25]–[29]. Further, IL-12 diminishes IL-2 expression by CD4 T cells [30]. Initially, we showed that IL-2 expression by Tconvs was modestly reduced after 48 hr with more substantial effects noted after 72 hr of IL-12 treatment (Fig. 8A). More strikingly, IL-2 production by CD8 T cells was greatly reduced after both 48 and 72 hr (Fig. 8A). In agreement with this diminished expression, levels of IL-2, measured by ELISA, were reduced in IL-12-treated cultures at 48 hr (Fig. 8B). T cell proliferation is augmented by IL-2, especially in tissues at sites of inflammation [31]–[33], yet the results in Fig. 1 show that Tconv proliferation is increased by IL-12 treatment. One possible explanation for these results is that IL-12 preferentially induces IL-2R expression on Tconvs; IL-12 was previously shown to enhance IL-2R expression on CD8 T cells [34]. As shown in Fig. 9 (A–B, D–E), this is indeed the case. Treatment with IL-12 in the presence of anti-CD3 mAb induced up-regulation of both the α (CD25) and β (CD122) chains of the IL-2R on Tconv and CD8 T cells in a dose-dependent manner. In contrast, IL-12 treatment diminished CD25 expression on Tregs while not affecting CD122 expression, reducing the ability of Tregs to compete for IL-2 in the culture. Of note, even though IL-12 induced increased IFN-γ expression by Tconv and CD8 T cells, IFN-γ, by itself, was unable to induce diminished expression of IL-2 (Fig. 8B) or IL-2R upregulation on non-Treg T cells (Fig. 9C).

As shown in Fig. 1, one of the effects of IL-12 treatment was decreased Foxp3 expression by Tregs. Previous studies showed that IL-2 is required for maximal Foxp3 expression [35] but whether IL-12-mediated reduction in Foxp3 expression occurred through effects on IL-2 signaling was not examined. To address the role for IL-2 signaling in IL-12-mediated effects, we treated cells with exogenous IL-2. IL-2 treatment partially reversed the effect of IL-12, resulting in higher levels of Foxp3 in Tregs (Fig. 9F).

### IL-27 Diminishes IL-2 Expression but does not Increase IL-2R Expression on Tconv and CD8 T Cells

**Figure 10:**
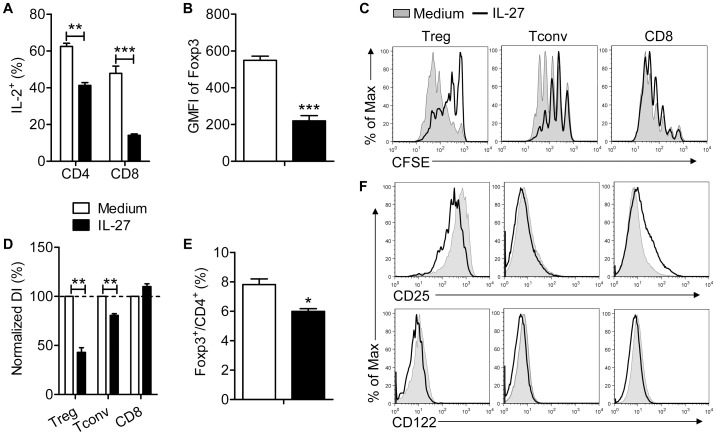
IL-27 inhibits IL-2 production, but only modestly affects IL-2R expression by Tconv and CD8 T cells. Lymphocytes were prepared from naïve B6 mice and treated with anti-CD3 mAb and IL-27 (100 ng/ml) for 66 hr. (A) The fraction of CD4 and CD8 T cells expressing IL-2 was diminished. Data are from 3 independent experiments. (B) IL-27 diminished Foxp3 GMFI in Tregs. Samples were analyzed in triplicate. Data are from one experiment representative of 3 independent experiments. (C, D, E) Lymphocytes were labeled with CFSE prior to incubation. IL-27 reduced proliferation of Tregs and Tconvs but not CD8 T cells (C, D), resulting in a modest decrease in the percentage of Foxp3^+^ Tregs (E). Data are from 3 independent experiments. (F) IL-27 treatment resulted in small changes in CD25 expression on Tregs and CD8 T cells but not Tconvs and in a small decrease in CD122 expression on all three cell types. Data are representative of 3 independent experiments. *P<0.05, **P<0.01, ***P<0.001, Student’s two-tailed un-paired *t* tests (A, B, E) or paired *t* tests (D).

IL-27 inhibits Treg development *in vivo* and decreases IL-2 expression by CD4 T cells *in vitro* [30]. To determine whether IL-27 has similar effects as IL-12, we treated lymphocyte cultures with anti-CD3 mAb and IL-27 or medium. Similarly to IL-12, IL-27 diminished IL-2 production by Tconvs and CD8 T cells (Fig. 10A) and down-regulated Foxp3 expression by Tregs (Fig. 10B). However, unlike IL-12, IL-27 decreased proliferation of Tconvs and Tregs and had no effect on CD8 T cell expansion (Fig. 10C, D) resulting in a relatively slight decrease in Treg frequency (Fig. 10E). The effects of IL-12 were mediated both by diminished IL-2 expression and changes in the relative amounts of IL-2R on Tregs and Tconvs and CD8 T cells. IL-27 reduced CD25 and CD122 expression on Tregs but only marginally affected expression on non-Tregs (Fig. 10F). Collectively, these results suggest that Tregs are better able to compete for IL-2 in IL-27 compared to IL-12-treated cultures.

## Discussion

A previous report, using co-cultures of purified Tconvs and Tregs, showed that IL-12 could relieve Treg-mediated suppression of Tconv proliferation and activation [12]. Here, we extend these observations to analyze the effects of IL-12 on T cells in unfractionated lymphoid cultures and identify a basis for at least some of the effects of IL-12. We show that IL-12 decreases IL-2 production by Tconv and CD8 T cells and differentially modulates IL-2R expression on Tregs and non-Tregs. IL-2R is up-regulated on Tconv and CD8 T cells and down-regulated on Tregs. When coupled with decreased IL-2 production, this results in a milieu that favors non-Treg proliferation. The effect of IL-12 on CD25 expression by CD8 T cells is especially notable. In the absence of IL-12, levels of CD25 are much greater on Tregs than CD8 T cells, but treatment with IL-12 at 1 ng/ml results in levels of CD25 on CD8 T cells that exceed those on Tregs in the same culture (Fig. 8B, C). Levels on CD8 T cells are substantially higher than those on Tconv cells, explaining why IL-12 more readily relieves Treg-mediated suppression of CD8 T cell proliferation. These results are consistent with previous reports showing that IL-2R is down-regulated on Tregs in inflamed tissues in the setting of infectious or autoimmune diseases and that IL-2 levels are reduced at these sites, resulting in the outgrowth of non-Treg T cells [5], [36], [37].

One probable consequence of these changes in IL-2R expression, in conjunction with IL-12-mediated decreases in IL-2 production, is that Tregs are less able to compete for the smaller amount of IL-2 present in the culture. These changes result in diminished Treg proliferation and perhaps decreased Treg suppressive function on a per cell basis (as measured by decreased Foxp3 expression (Fig. 1E)), resulting in decreased total Treg suppressive capacity in the culture.

The importance of IL-2 for Treg function is well established [29], [35], [36] and has been confirmed in clinical studies. In patients with either hepatitis C virus-induced vasculitis or graft versus host disease, treatment with low doses of IL-2 therapy increased Treg numbers and decreased clinical disease [38], [39]. These results are also consistent with others showing that Treg numbers were increased when cancer patients were treated with low dose IL-2 [40], [41]. Exogenously administered IL-2 also corrects IL-12-mediated decreases in Foxp3 levels in Tregs (Fig. 8B); whether low level IL-2 treatment also increases Foxp3 levels in Tregs in patients, in addition to increasing Treg numbers, has not yet been addressed.

Further support for an important role for IL-2R levels on Tconv and CD8 T cells comes from studies of IL-27. IL-27 is a cytokine with pro- and anti-inflammatory properties [42]–[45]. Mice transgenic for the expression of IL-27 exhibit diminished IL-2 production, which results in decreased numbers of Tregs and subsequently, a systemic inflammatory disease [46]. We showed that IL-27 diminished IL-2 expression, consistent with these results, but IL-27 only modestly decreased Treg frequency (Fig. 9D). Consistent with a role for IL-2R expression on non-Tregs, there were minimal changes in IL-2R levels on these cells after IL-27 treatment (Fig. 9E). Thus, under these conditions, Tregs were presumably able to compete for IL-2 even in the presence of reduced amounts of IL-2.

Another consequence of IL-12 treatment is the induction of IFN-γ expression by Tregs. Tregs express IFN-γ at sites of inflammation under conditions of Th1-type lethal and non-lethal infections [5], [8]. It has been difficult to determine whether IFN-γ affects Treg suppressive function, since Tregs in these settings include both IFN-γ^+^ and IFN-γ^-^ cells. Here we showed that IFN-γ expression did not diminish Treg suppressive function. These results are in agreement with others showing that *in vitro*-generated IFN-γ^+^ and IFN-γ^-^ Tregs were equally suppressive in a mouse model of colitis [13]. In other experimental settings, loss of Foxp3 and gain of inflammatory cytokine expression suggested that Tregs converted into effector T cells [22], [47]. If Tregs at sites of inflammation can generally express IFN-γ and still remain suppressive, these results raise the possibility that IFN-γ^+^ Tregs, instead of entering the effector T cell pool, could lose IFN-γ and revert to a classic Treg phenotype. Whether these cells would preferentially re-express IFN-γ on repeat exposure to antigen remains to be determined.

Several studies including this one demonstrate an important role for IL-12 in IFN-γ expression by Tconv and CD8 T cells and Tregs *in vitro* [5], [13]. Further, IL-12 is critical for IFN-γ production by Tregs in mice with autoimmune colitis [13]. However, the requirement for IL-12 in IFN-γ expression by Tconv and CD8 T cells or Tregs in virus-infected animals is less clear. To investigate whether IL-12 signaling is critical for IFN-γ production by Tregs in virus-infected mice, we infected IL-12Rβ2^−/−^ mice with the mildly neurovirulent rJ2.2 strain of MHV [48]. We detected slightly lower frequencies of virus-specific IFN-γ expressing Tregs and Tconv in IL-12Rβ2^−/−^ mice, consistent with a previous report showing that IL-12 modestly decreased the frequencies of virus-specific T cells in MHV-infected IL-12p35^−/−^ mice [49]. The absence of IL-12 signalling may not have had a substantial effect because we detected no differences in CD25 expression on Tregs and virus-specific non-Treg T cells when cells harvested from the brains of IL-12Rβ2^−/−^ mice and B6 mice were compared (data not shown). Our results are also consistent with others showing IFN-γ production was relatively normal in IL-12^−/−^ or IL-12R^−/−^ mice infected with viruses such as lymphocytic choriomeningitis virus, influenza A virus, adenovirus or hepatotropic strains of MHV. In contrast, IL-12 was essential for IFN-γ expression in mice infected with some intracellular non-viral pathogens, such as Leishmania species and Toxoplasma gondii [50].

In summary, our results show that Treg-mediated suppression of T cell proliferation, especially that of CD8 T cells is nearly ablated in the presence of IL-12. IL-12 induces IFN-γ expression by Tregs, but this does not affect Treg immunosuppressive function. Rather, the ability of IL-12 to reverse Treg immunosuppression results, at least in part, from effects on IL-2 and IL-2R expression and by extension, differential IL-2 signaling in mixed cultures of lymphocytes.
